# The Utility of Novel Kidney Injury Biomarkers in Early Detection of CSA-AKI

**DOI:** 10.3390/ijms232415864

**Published:** 2022-12-14

**Authors:** Jakub Udzik, Aleksandra Waszczyk, Iwona Wojciechowska-Koszko, Paweł Kwiatkowski, Paulina Roszkowska, Karolina Rogulska, Krzysztof Safranow, Andrzej Biskupski, Sebastian Kwiatkowski, Ewa Kwiatkowska

**Affiliations:** 1 Department of Cardiac Surgery, Pomeranian Medical University, Powstancow Wielkopolskich 72, 70-111 Szczecin, Poland; 2 Department of Infectious Diseases, Hepatology and Liver Transplantation, Pomeranian Medical University, Arkonska 4, 71-455 Szczecin, Poland; 3 Department of Diagnostic Immunology, Pomeranian Medical University, Powstancow Wielkopolskich 72, 70-111 Szczecin, Poland; 4 Department of Biochemistry and Medical Chemistry, Pomeranian Medical University, Powstancow Wielkopolskich 72, 70-111 Szczecin, Poland; 5 Department of Obstetrics and Gynecology, Pomeranian Medical University, Powstancow Wielkopolskich 72, 70-111 Szczecin, Poland; 6 Department of Nephrology, Transplantology and Internal Medicine, Pomeranian Medical University, Powstancow Wielkopolskich 72, 70-111 Szczecin, Poland

**Keywords:** CSA-AKI, kidney injury biomarkers, cardiac surgery, cardiopulmonary by-pass

## Abstract

Cardiac surgery-associated acute kidney injury (CSA-AKI) is one of the most common complications of cardiac surgery procedures. In this study, the authors attempt to provide new data regarding the application of novel kidney injury biomarkers in the early diagnostics of CSA-AKI. 128 adult patients undergoing elective cardiac surgery procedures with the use of cardiopulmonary by-pass (CPB) were enrolled in this study. Novel kidney injury biomarkers were marked in the plasma and urine 6 h after weaning from the CPB. A significant difference in the postoperative biomarkers’ concentration between the AKI and no-AKI group was found, regarding plasma IL-8, plasma TNF-α and urine NGAL, normalized for creatinine excretion (NGAL/Cr). These were also independent predictors of CSA-AKI. An independent risk factor for CSA-AKI proved to be preoperative CKD. Plasma IL-8 and TNF-α, as well as urine NGAL/Cr, are independent early indicators of CSA-AKI and pose a promising alternative for creatinine measurements. The cut-off points for these biomarkers proposed in this investigation should be confronted with more data and revised to achieve a suitable diagnostic value.

## 1. Introduction

Cardiac surgery-associated acute kidney injury (CSA-AKI) is one of the most common complications of cardiac surgery procedures, affecting approximately 25–30% of patients [1,2]. The KDIGO guidelines for diagnostics of acute kidney injury (AKI) published in the 2012 [3,4] are still up to date, as they are based on a highly standardized and repetitive measurement—serum creatinine. Up to this day, in routine clinical practice, physicians rely on serum creatinine in the diagnostics of CSA-AKI as well due to the lack of a superior alternative. There are constant attempts, however, to find such an alternative. Many researchers aim to find more specific markers of kidney injury with more favorable serum kinetics and those unaffected by cardiac surgery’s specific features [5,6,7,8].

The main issue concerning CSA-AKI diagnostics with serum creatinine is the massive fluid transfusion, mainly during cardiopulmonary by-pass. This involves a priming fluid of approximately 1500 mL, at least 500 mL of cardioplegic solution (assuming the use of lower volume blood cardioplegia) and intravenous fluids from anesthesia, which rarely account for less than 500 mL. This sums to at least 2500 mL of fluids administered to the patient during the operation. Additionally, there are fluids administered in the postoperative period. As a result of this considerable fluid intake, serum metabolites (including creatinine) become diluted. For this reason, it usually takes about 24 h for the serum creatinine to accumulate and meet the AKI criteria [9,10]. Studies conducted in recent years prove it is possible to detect CSA-AKI as soon as 6 h after weaning from cardiopulmonary by-pass (CPB) using urine kidney injury biomarkers, such as neutrophil gelatinase-associated lipocalin (NGAL), kidney injury molecule 1 (KIM-1), matrix metalloproteinase 9 (MMP-9) and interleukin 18 (IL-18) [6,8,11,12,13,14]. Additionally, the molecules present in the serum, such as interleukin 6 (IL-6), interleukin 8 (IL-8) and tumor necrosis factor-alpha (TNF-α), are a promising diagnostic tool in this regard [8,15,16,17].

IL-6 is produced by myeloid cells together with interleukin 1 beta (IL-1 β) and TNF-α, which leads to an increase in IL-6 production through a positive feedback mechanism. This mechanism is so potent that the IL-6 level can increase as much as six orders of magnitude. This makes IL-6 a sensitive indicator of inflammation, infection and neoplasia growth [18,19,20].

IL-8 is another proinflammatory cytokine produced by mononuclear macrophages in systemic infections. Its increase is associated with higher mortality in patients with sepsis [21]. It is also known to play a role in the progression of acute inflammatory changes within the kidney to the chronic form [22].

One of the main pathophysiological mechanisms underlying AKI is renal ischemia followed by reperfusion (ischemia–reperfusion injury—IRI). The most important cytokine in this process is TNF-α, which acts by activating cyclooxygenase 2 (COX-2) [23]. The major role of TNF-α was proved by studies that demonstrated a kidney protective influence of anti-TNF-α therapy in ischemic AKI [24].

NGAL is secreted by the cells of the proximal kidney tubule, the ascending limb of the Henle loop and the collecting duct. NGAL, present in urine, is produced in the lungs, liver and leukocytes (including leukocytes that infiltrate kidneys) as a response to the inflammation process [25], which is a disadvantage of this biomarker. Nevertheless, combined with other specific renal biomarkers (such as KIM-1), urine NGAL concentrations may provide additional information about the extent of kidney tissue damage. After kidney injury, NGAL reaches its maximal urine concentration in 6 h, and its prolonged presence is linked to tissue regeneration processes within the kidney [11].

KIM-1 is an orphan transmembrane receptor produced in substantial amounts in the proximal tubule subsequent to an injury [26]. It can be detected in urine a few hours after kidney injury, and it is proven to have a high sensitivity in detecting AKI. After reaching its maximal urine concentration within 72 h from injury, it maintains elevated, stimulating proximal tubule cell regeneration [12,27].

MMP-9 is dynamically expressed and activated in inflamed tissues, degrading the extracellular matrix proteins and taking part in tissue remodeling secondary to injury [28,29]. MMP-9 is also related to urine KIM-1 elevation, as it mediates the shedding of the extracellular domain of this receptor [30].

IL-18 is produced as an inactive precursor by macrophages, dendritic cells, epithelial cells, keratinocytes, chondrocytes and fibroblasts. In the kidneys, its primary sources are tubule epithelial cells [31,32,33]. An IL-18 increase resulting from AKI was demonstrated in numerous clinical studies, including studies on patients after kidney transplant who developed acute tubular necrosis [34,35]. Urine IL-18 concentration is reported to increase 4–6 h after cardiac surgery [13].

The above-described biomarkers were incorporated into this investigation, as they have dynamic serum kinetics (IL-6, IL-8 and TNF-α) or are secreted with urine (NGAL, KIM-1, MMP-9 and IL-18), which makes them a noninvasive alternative to serum creatinine measurements. All of them were also proved to be reliable kidney injury indicators. An early diagnosis is vital for the rapid treatment of CSA-AKI and, as a consequence, for reducing morbidity and mortality due to postoperative renal failure [4,27,36,37,38,39,40]. In this study, the authors attempt to provide new data regarding the application of novel kidney injury biomarkers in the diagnostics of CSA-AKI.

## 2. Results

The overall prevalence of CSA-AKI in this study population was 32%. Most AKI cases were diagnosed 24 h after the operation (63.41%). After the 24th postoperative hour, the number of newly diagnosed AKI gradually decreased: 17.07% on the 2nd postoperative day, 12.2% on the 3rd and 7.32% in the next 48 h.

The preoperative characteristics of the study population with regard to AKI occurrence are included in Table 1.

The overall preoperative incidence of chronic kidney disease (CKD) in this study population was 17.19%, out of which 68.18% was KDIGO 3a stage, and the remaining 31.82% was KDIGO 3b stage.

In the AKI group, there was a higher percentage of patients who underwent complex procedures (coronary artery by-pass grafting combined with valvular surgery): 26.83% (vs. 10.34% in the control group, *p* = 0.034). The percentage of patients who underwent isolated coronary artery by-pass grafting and isolated valvular surgeries did not differ significantly between the groups (*p*-value 0.259 and 0.349, respectively). There was no difference between the total CPB time and aortic cross-clamp time between the AKI and no-AKI group (*p*-value 0.267 and 0.308, respectively).

**Figure 1:**
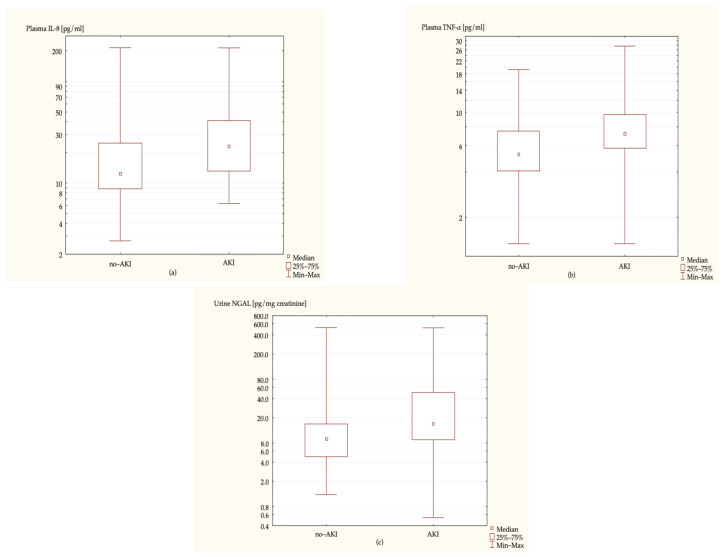
Postoperative biomarker concentrations in the control group and the AKI group (*p*-value < 0.001 in all cases). The Y-axis was logarithmized for improved data clarity. (**a**) Plasma IL-8 (pg/mL), (**b**) plasma TNF-α (pg/mL), (**c**) urine NGAL normalized for creatinine excretion (ng/mg).

A significant difference in the postoperative biomarkers’ concentration between the AKI and no-AKI group was found regarding plasma IL-8, plasma TNF-α and urine NGAL normalized for creatinine excretion (NGAL/Cr); *p*-value was <0.001 in all cases—Figure 1.

A positive correlation was observed between the patients’ age and some biomarker concentrations. Additionally, a negative correlation existed between some of the biomarkers and preoperative kidney function, as well as the mean hematocrit value during CPB—Table 2.

## 3. Discussion

Multivariable logistic regression was applied to adjust the analysis for potentially confounding factors (age, sex and the incidence of preoperative CKD). According to this analysis, the independent predictors of CSA-AKI were high plasma TNF-α, high plasma IL-8 and high urine NGAL/Cr. An independent risk factor for CSA-AKI proved to be preoperative CKD. There was a positive correlation between plasma IL-8 and plasma TNF-α, hence the loss of statistical significance for these biomarkers when incorporating them into one logistic regression model. For this reason, two different logistic regression models were calculated, as presented in Table 3.

**Figure 2:**
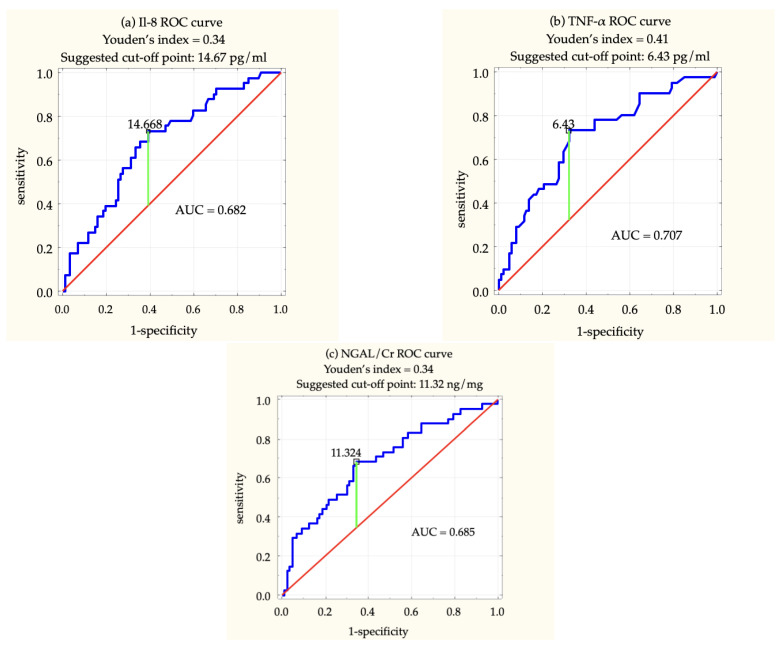
ROC analysis of the selected AKI indicators: (**a**) plasma IL-8, (**b**) plasma TNF-α and (**c**) urine NGAL normalized for creatinine excretion—NGAL/Cr. AUC—area under a curve.

Receiver operating characteristic (ROC) analysis was conducted to assess the biomarkers’ potential to serve as a diagnostic tool. Plasma IL-8, plasma TNF-α and urine NGAL/Cr proved to be the most suitable AKI indicators (AUC > 0.5, *p*-value < 0.001). The results of the analysis are presented in Figure 2.

Cardiac surgery procedures are burdened with considerable perioperative risk (2.2% overall mortality rate vs. 1.1% for non-cardiac surgeries in Europe and the USA) [41,42]. Considering the high incidence of CSA-AKI and its impact on patient survival, there is a need for an improved diagnostic strategy regarding this complication.

Numerous studies report elevated levels of novel kidney injury biomarkers in patients with postoperative AKI [6,12,16,28,43,44,45,46]. The data, however, are incoherent as different diagnostic criteria were used and different methods were applied in various studies. For instance, Wagener et al. [47], as well as Che et al. [48], assumed the RIFLE criteria for CSA-AKI diagnostics, while the golden standard nowadays is the KDIGO criteria. Wagener et al. also did not adjust the urine NGAL concentration to urine creatinine excretion, the same as Heise et al. [49], which is also not up to the current norms. Meta-analysis of the urinary NGAL performance in CSA-AKI diagnostics conducted by Zhou et al. [50] is naturally more comprehensive than this investigation, and yet, it merges data obtained from adult and child populations. This may influence the cut-off values given in that article as there are distinctive differences in the kidney injury biomarkers’ origin in the pediatric population [51]. A recent systematic review by Pan et al. [52] on the biomarkers’ accuracy in a hospital-acquired AKI prediction was not limited to cardiac surgery patients, which may influence the result’s usefulness in the field of cardiac surgery.

In this study, the authors wanted to provide transparent, up-to-date data concerning adult post-cardiac surgery patients, and so, all the current standards in this field of investigation were kept (available in the “Materials and Methods” section).

Despite a modest sample size, the authors provided cut-off points for the most promising biomarkers as a step toward the clinical application of this research. The Luminex^®^ technology used in this investigation was proven to have high reliability in the field of kidney injury research [53]. It is, therefore, reasonable to assume that researchers conducting similar investigations will also rely on this technology. The results of such investigations should be comparable to the results of this study, thus, providing an opportunity to merge these data.

As demonstrated by Youden’s index (<0.5 in all cases), the results of this study cannot be yet introduced to clinical practice. A larger patient cohort is required to improve the sensitivity and specificity of these tests. However, considering the favorable kinetics of these biomarkers and less invasive testing methods compared to serum creatinine (regarding NGAL), further endeavors in this field are highly recommended. In more than half of this study population, AKI could have been diagnosed up to four times faster if biomarkers were used instead of creatinine measurements. In the rest of the patients, the diagnosis could have been made even so much as days earlier. This lost time is a severe handicap for the kidneys striving to regenerate from injury, as it delays introducing a proper treatment.

Two essential issues concerning introducing novel biomarkers to routine diagnostics are poor testing standardization and high cost. Nonetheless, these obstacles can be overcome if sufficient data are gathered and the testing becomes more widespread.

While the main objective of this study was to provide new data regarding novel kidney injury biomarkers, other observations were made during this research. Older age, lower intraoperative hematocrit value and poorer preoperative kidney function are all known risk factors for CSA-AKI. In this study the authors demonstrated that they also directly correlate with the postoperative biomarkers’ concentrations. This suggests that these factors enhance kidney damage during cardiac surgery and should not be underestimated even in the absence of clinically evident AKI.

## 4. Materials and Methods

A cohort of 128 adult patients undergoing elective cardiac surgery procedures with the use of CPB were enrolled in this study over a 24-month period. Regional Bioethical Committee approved the study protocol and conditions (document’s signature: KB-0012/45/2021). Informed consent was obtained from each patient prior to the enrollment.

Inclusion criteria:

- Qualification for an elective cardiac surgery procedure with the use of CPB;
- Written consent for study enrolment.

Exclusion criteria:

- End-stage renal disease (eGFR < 15 mL/min/1.73 m^2^);
- Kidney artery stenosis in medical history;
- Active inflammatory disease;
- Active neoplasm;
- Mayor intraoperative complications causing hypotension;
- Mayor postoperative complications causing hypotension;
- Need for continuous renal replacement therapy (CRRT) within 6 h after the procedure.

Blood and urine samples were collected from the patients 6 h after weaning from CPB. Blood was collected from the radial artery using S-Monovette 3.4 mL sterile containers (K3 EDTA: 1.6 mg/1 mL of blood; SARSTEDT AG & Co. KG Sarstedtstrasse 1, 51588 Numbrecht, Germany). Urine was collected directly from the Foley catheter using standard non-sterile urine containers. After the collection, blood and urine samples were stored at 5 °C for no longer than 4 h and subsequently centrifuged (4 °C, 10 min, 4000 RPM). After centrifugation, 1 mL of supernatant was taken and stored at −70 °C for no longer than six months.

Quantitative assessment of IL-6, IL-8 and TNF-α (in plasma), as well as NGAL, KIM-1, IL-18 and MMP-9 (in urine) levels in patients enrolled in this study was performed using Luminex xMAP technology (Luminex Corporation, Austin, TX, USA). The first measurements using urine samples were carried out on the native urine form, and the results obtained exceeded the upper detection limit for this assay. Due to that, an optimization process was conducted in order to determine the optimal dilution for all samples. The following dilutions were selected as optimal: 1:2 (for NGAL and KIM-1) and 1:10 (for IL-18 and MMP-9). Plasma samples were tested in their native form. Thawed samples were centrifuged (4 °C, 4 min, 16,000 RPM) immediately before use. The analysis was conducted according to manuals provided with the multiplex reagent kit (Human Magnetic Luminex Assay, R&D Systems, Minneapolis, MN, USA) using the Luminex 100 analyzer and Xponent 4.2 Build software. On a microplate, 50 μL of samples were incubated in each well with a 50 μL diluted microparticle cocktail for 2 h at room temperature on a horizontal orbital microplate shaker (0.12” orbit) set at 800 ± 50 RPM. A magnetic Manual MagPlex^®^ Bead Washing device was used to wash the samples. The microplates were placed on the separator for 1 min, after which the remains not attached to the magnet were removed. Subsequently, 100 μL of the wash buffer was added to each well and incubated for 1 min, after which the remains not attached to the magnet were once again removed. This last step was repeated three times.

In this step, 50 μL of a diluted Biotin-Antibody Cocktail were then added to the wells and incubated for 1 h at room temperature on the shaker (800 ± 50 RPM). Microplates were washed again. In this step, 50 μL of diluted Streptavidin-PE were added and incubated for 30 min at room temperature on the shaker (800 ± 50 RPM). Microplates were washed again. The microparticles were then resuspended by adding 100 μL of the wash buffer and incubated for 2 min on the shaker (800 ± 50 RPM). The microplates were read within 90 min using the Luminex^®^ standards. Concentrations of the analyzed biomarkers were calculated based on a standard 6-point curve.

Creatinine concentration was also determined in the urine samples to normalize the urinary biomarkers’ concentrations to creatinine excretion. Creatinine was measured in the defrosted urine samples after 12 h of storage in a dark and cooled compartment (5 °C). A kinetic colorimetric test based on Jaffe’s method was used to measure the creatinine concentration in urine (Cobas Pro c503 module, Roche, Basel, Switzerland).

A quantitative assessment of the protein level in the urine samples was not conducted as there was, at most, a trace amount of protein present in the samples. Such an amount of protein was deemed insignificant as it does not interfere with the biomarker’s level measurement. Postoperative AKI was diagnosed according to the KDIGO criteria (≥0.3 mg or 50% increase in serum creatinine within 48 h or diuresis < 0.5 mL/kg/h for at least 6 h) until the 5th postoperative day. Serum creatinine was measured on the day preceding the operation, on the 1st postoperative day and, subsequently, every 48 h.

Fisher’s exact test was used to compare qualitative variables between the groups. Since most quantitative variables were non-normally distributed, they were presented as median (M) and interquartile range (Q1–Q3). Following, non-parametric tests were used: Mann–Whitney test for differences between the groups and Spearman’s rank correlation coefficient for correlations between the parameters. Multivariable logistic regression was used to find independent predictors of AKI with normalizing logarithmic transformation of biomarker concentrations. Receiver operating characteristic (ROC) analysis was conducted to estimate the diagnostic value of the biomarkers in relation to AKI prognosis. The suggested cut-off points were based on the maximization of Youden’s index. Associations with *p* < 0.05 were considered statistically significant. Calculations were performed with Statistica 13 program.

The objective of this study was to assess the utility of novel kidney injury biomarkers in the early diagnostics of CSA-AKI.

## 5. Conclusions

Plasma IL-8 and TNF-α, as well as urine NGAL/Cr, are independent early indicators of CSA-AKI and pose a promising alternative for creatinine measurements. Cut-off points for these biomarkers proposed in this investigation should be confronted with more data and revised to achieve a suitable diagnostic value. Future investigations in this area should focus on providing specific data to improve these biomarkers’ clinical performance. 

## Figures and Tables

**Table 1:** Preoperative characteristics of the study population.

	AKI (*n* = 41)	no-AKI (*n* = 87)	*p*-Value
Age M (Q1–Q3) (years)	70 (67–75)	67 (61–72)	**0.013**
Male *n* (%)	25 (60.98)	65 (74.71)	0.147
Female *n* (%)	16 (39.02)	22 (25.29)	
BMI M (Q1–Q3)	27.85 (25.91–30.70)	28.09 (25.53–31.10)	0.815
ES II M (Q1–Q3)	3.81 (2.10–5.42)	2.92 (1.70–4.77)	0.267
Hypertension *n* (%)	35 (85.37)	67 (77.01)	0.349
Diabetes *n* (%)	15 (36.59)	27 (31.03)	0.550
Dyslipidemia *n* (%)	19 (46.34)	38 (43.68)	0.850
CKD *n* (%)	15 (36.59)	7 (8.05)	**<0.001**
Preoperative hematocrit (Q1–Q3) (%)	39.50 (37.35–42.80)	41.40 (39.30–43.20)	**0.045**
Hb_A1C_ M (Q1–Q3) [%]	5.90 (5.70–6.70)	5.90 (5.50–6.30)	0.344
Preoperative creatinine (Q1–Q3) (mg/dl)	1.02 (0.89–1.26)	0.89 (0.76–1.01)	**0.002**
Preoperative eGFR Q1–Q3) (mL/min/1.73 m^2^)	65 (55–81)	82 (71–93)	**<0.001**

Legend: BMI—body mass index, CKD—chronic kidney disease, eGFR—estimated glomerular filtration rate, ES II—EuroSCORE II, Hb_A1C_—glycated hemoglobin and M—median.

**Table 2:** Spearman’s correlation coefficient analysis.

	R	*p*-Value
Age and TNF-α	0.234	**0.008**
Age and NGAL/Cr	0.238	**0.007**
Preoperative eGFR and IL-8	−0.190	**0.031**
Preoperative eGFR and TNF-α	−0.373	**<0.001**
Preoperative eGFR and NGAL/Cr	−0.226	**0.010**
Mean HtCPB and IL-8	−0.251	**0.004**

Legend: eGFR—estimated glomerular filtration rate, HtCPB—hematocrit value during CPB, IL-8—plasma IL-8 concentration 6 h after weaning from CPB, NGAL/Cr—urine NGAL concentration 6 h after weaning from CPB normalized for creatinine excretion and TNF-α—plasma TNF-α concentration 6 h after weaning from CPB.

**Table 3:** Logistic regression models for independent CSA-AKI predictors and risk factors.

(a) Logistic Regression Model Involving Age, Sex, Preoperative CKD, Plasma TNF-ɑ and Urine NGAL/Cr			
	OR	CI	*p*-Value
Age	1.03	0.97–1.09	0.377
Sex	0.94	0.34–2.59	0.910
Preoperative CKD	3.64	1.19–11.16	**0.022**
Log TNF-ɑ	3.19	1.20–8.49	**0.019**
Log NGAL/Cr	1.68	1.15–2.47	**0.007**
**(b) Logistic Regression Model Involving Age, Sex, Preoperative CKD, Plasma IL-8 and Urine NGAL/Cr**			
	**OR**	**CI**	***p*-Value**
Age	1.04	0.98–1.10	0.230
Sex	1.16	0.42–3.23	0.774
Preoperative CKD	5.03	1.71–14.78	**0.003**
Log IL-8	1.88	1.07–3.30	**0.026**
Log NGAL/Cr	1.57	1.08–2.29	**0.018**

Legend: CI—95% confidence interval, CKD—chronic kidney disease, Log NGAL/Cr—log-transformed urine NGAL concentration 6 h after weaning from CPB normalized for creatinine excretion, Log TNF-ɑ—log-transformed plasma TNF-ɑ concentration 6 h after weaning from CPB and OR—odds ratio, Log IL-8—log-transformed plasma IL-8 concentration 6 h after weaning from CPB and OR—odds ratio.
